# Cranial structure and condylar asymmetry of adult patients with rheumatoid arthritis

**DOI:** 10.1186/s12903-023-03001-2

**Published:** 2023-05-09

**Authors:** Maria Grazia Piancino, Rossana Rotolo, Rosangela Cannavale, Giovanna Cuomo, Francesco Masini, Paola Dalmasso, Fabrizia D’Apuzzo, Letizia Perillo, Ludovica Nucci

**Affiliations:** 1 Department of Surgical Sciences-Orthodontic Division, Italian Research Center, University of Turin, Turin, Italy; 2 Multidisciplinary Department of Medical-Surgical and Dental Specialties, University of the Study of Campania Luigi Vanvitelli, Naples, Italy; 3 Department of Precision Medicine, University of the Study of Campania Luigi Vanvitelli, Naples, Italy; 4 Department of Medical and Surgical Sciences, University of the Study of Campania Luigi Vanvitelli, Naples, Italy; 5 Department of Public Health and Paediatrics, University of Turin, Turin, Italy

**Keywords:** Arthritis, rheumatoid, Cephalometry, Orthopantomography, Mandibular condyle, Facial asymmetry, Temporomandibular joint

## Abstract

**Objective:**

The aim of this prospective cross sectional study was to evaluate the cranial structure and condylar asymmetry of adult patients with rheumatoid arthritis (RA) diagnosed after 25 years of age compared to a healthy adult control group.

**Methods:**

Eighteen adult patients (57.4 ± 11.4 years) with RA were compared with a control group. Cephalometric analysis and the Habets method for the calculation of the condylar asymmetry were used. The main cephalometric data investigated were focused on the diagnosis of hyperdivergent cranial structure (NSL/ML, NL/ML), backwards rotation of the mandible (Fh/ML), short vertical ramus (Ar:Go), steep mandibular plane (ML/Oc).

**Results:**

The cephalometric data considered were not significantly different in the RA vs controls except for the steepness of the occlusal plane (NL/Oc), which was steeper in the patients group (*P* < 0.02) and the ramus of the mandible which was greater in patients. The asymmetry of the condyles was significant (*P* < 0.003) and different from the control group, but that of the ramus was not.

**Conclusions:**

In this study, RA patients diagnosed after 25 years of age did not show a different pattern of growth with respect to the control group. As expected, the condyles showed a difference being asymmetrical in RA patients due to the high turnover of this joint reacting to severe systemic inflammation in conditions of continuous functional work, load and forces. This study follows a previous study with the same research plan conducted on young JIA patients who showed a different pattern of growth of the skull leading to a severe hyperdivergent cranial structure with backward rotation of the mandible; this is mainly due to the insufficient growth of the condylar site exposed to the inflammatory process during development. Unlike JIA patients, this study showed that RA patients follow an individual growth pattern not affected by inflammation, even if they show joint asymmetry.

## Introduction

Some medical conditions, such as rheumatoid arthritis (RA) may severely affect the normal functions of temporomandibular joints (TMJs) in adult patients [1–4]. Rheumatoid arthritis is a systemic and chronic autoimmune disease characterized by persistent inflammation at the synovial joints causing morphological deformities and pain influenced by genetic and environmental factors, the etiopathogenesis of which is still unknown [5–8]. Its prevalence is approximately 0.5-1 % in the whole population, with a higher incidence in females [9]. TMJ involvement in patients affected by RA has been reported in previous studies with a prevalence ranging from 45% to 92.8%. It follows the same destructive path occurring in other body joints in relation to the severity and duration of RA worsening by an unavoidable daily functional commitment [10–13]. Destruction of the cortical and subcortical bone can lead to almost complete loss of the normal condyle morphology [14]. The most common clinical signs and symptoms of TMJ involvement with arthritis are arthralgia, swelling, stiffness during mouth opening and, upon waking, joint noises and limited functions [9, 10]. Recently, it was found that 98.4% of RA patients presented alterations in some TMJ functions and 62.9% had a moderate or severe temporomandibular disorder (TMD) [15]. Their TMJ radiographic records showed abnormalities in the integrity of the bone cortex, bone erosions, asymmetry and flattening of the condyles, and joint space narrowing. Unfortunately, the presence of these TMJ alterations in patients with RA is often ignored and rarely reported in the patient’s history due to paucisymptomaticity; consequently, the treatment is focused on joints in which involvement is clinically more evident and the diagnosis of TMJ involvement arrives often too late to prevent permanent damage. Even if the patient does not report signs and symptoms of the inflammatory process, they should be carefully searched and early diagnosis, treatment and monitoring of TMJ disturbances should be considered [9, 16, 17]. In fact, a recent study assessed the prevalence of TMJ involvement and specific oral factors as predictive values in RA development [18]. In addition, Yıldırım et al. showed a relationship between radiographic temporomandibular joint (TMJ) changes and the disease activity score 28 (DAS28) in rheumatoid arthritis (RA) patients [14].

Previous studies evaluated condylar asymmetry and cranial features in young patients with juvenile idiopathic arthritis (JIA) showing a risky growth pattern of the skull and condyle when this pathology occurs during development [19–23]. To the best of our knowledge, these data (cephalometry and condyle) still need to be evaluated in adult patients with RA. It is intriguing to determine if they show the same anomalous pattern with unsteady vectorial functions as JIA patients. The hypothesis is that, when RA is diagnosed in adulthood at the end of cranial growth without the influence of pathological inflammation during development, the alterations of the skull and condyle previously shown in young JIA patients [19–23], should not be present. Thus, the aim of this study was to evaluate the cranial structure and condylar asymmetry of adult patients with rheumatoid arthritis (RA) compared to a control group.

## Materials and methods

This prospective cross sectional study was conducted on patients referred from rheumatologists at the Orthodontic Programs at the University of Campania Luigi Vanvitelli for orthodontic consultations from January 2019 through January 2021. The inclusion criteria were (1) a confirmed diagnosis of RA (diagnosis of RA after 25 years of age), (2) regular follow-up by a rheumatologist, and (3) dental panoramic and lateral skull radiographs after RA diagnosis for dental reasons. Patients were excluded if they had incomplete medical records, congenital or acquired facial anomalies, a history of facial fractures and previous maxillofacial surgery or orthodontic treatment. RA patients were classified as seropositive and seronegative based on whether the rheumatoid factor (RF) and/or anti-cyclic citrullinated peptide (anti-ccp) were positive. The patients with RA recruited were in treatment with nonsteroidal anti-inflammatory drugs (NSAIDs) or corticosteroids during the acute phase of the disease associated with disease-modifying antirheumatic drugs (DMARDs) such as hydroxychloroquine or methotrexate. The patients who did not have any benefit after this pharmacological treatment (“nonresponders”) and those with an initial severe prognosis of RA were treated with a biological drug. A control group with normal growth and occlusion with mild crowding, without JIA or RA, maxillofacial or TMJ disorders or previous orthodontic or surgical treatments and with no significant medical history was selected for the study.

The study was approved by the Ethics Committee of the University of Campania Luigi Vanvitelli, Italy (Prot N° 309) and informed consent was obtained from each patient before recruitment. All experiments were performed in accordance with the Declaration of Helsinki.

**Fig. 1:**
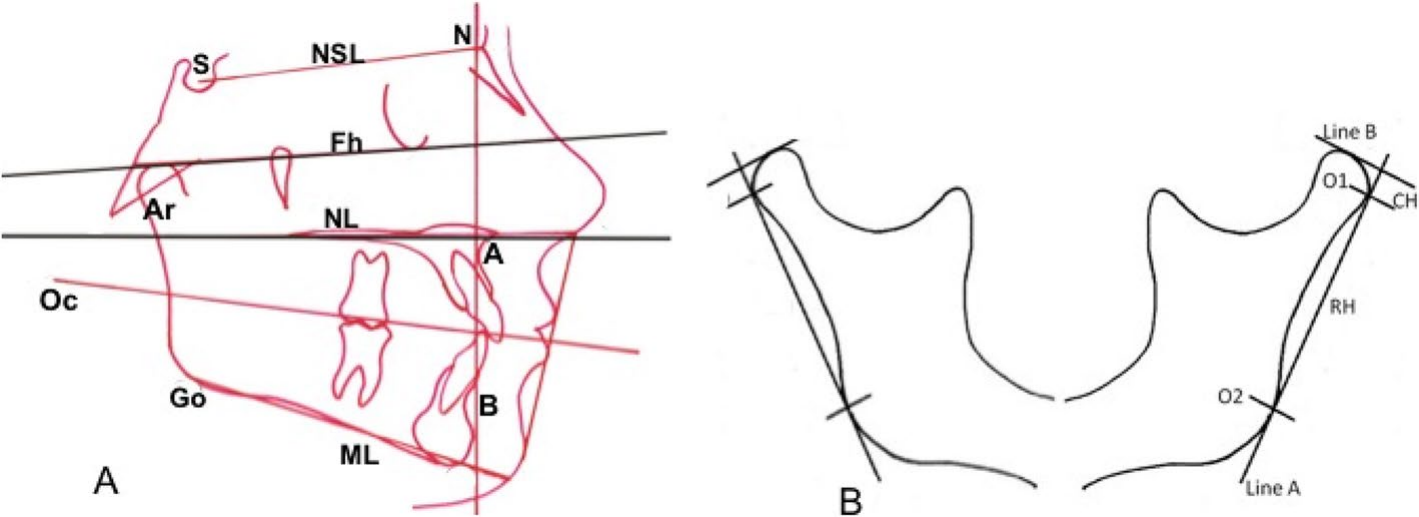
Panel **A** Cephalometric landmarks and planes; Panel **B** Method of Habets/Hansson used to evaluate condylar and mandibular asymmetry; see text, Materials and methods section

For every patient included in the protocol, a dental, orthodontic and orofacial diagnosis was performed clinically and through study dental casts and radiographs analyses. Specifically, the craniofacial structure was evaluated by cephalometric analysis of the lateral skull radiographs. Ten angular and three linear measurements (Fig. 1, panel A) were performed manually on acetate films on profile radiographs by the same trained operator to avoid interobserver variability. Angular measurements are: SNA, angle formed between sella, nasion, and point A, sagittal cranial relationship (the relationship in the sagittal plane between the cranial base as reference and the upper maxilla) according to Stainer; SNB, angle formed between sella, nasion, and point B, sagittal cranial relationship (the relationship in the sagittal plane between the cranial base as reference and the mandible) according to Stainer; ANB, angle formed between SNA and SNB planes, sagittal cranial relationship (the relationship in the sagittal plane between the cranial base and the mandibular plane); NSL/ML, angle formed between Sella-Nasion line and Mandibular Line, mandibular inclination relative to the cranial base (the relationship in the sagittal plane between the cranial base and the mandibular plane); Fh/ML, angle formed between Frankfurt plane and Mandibular Line, mandibular inclination relative to the Frankfurt plane (the relationship in the sagittal plane between the Frankfurt plane and the Mandibular plane); NL/ML, angle formed between Nasion Line and Mandibular Line (angle between superior maxilla(NL) and the body of the mandible(ML) to evaluate the mandibular divergency, according to Schudy; ArGo/ML, gonial angle formed between the ramus (ArGo) and the body (ML) of the mandible; NL/Oc, angle between superior maxilla(NL) and occlusal functional plane (OC), to evaluate the orientation of the occlusal plane; ML/Oc, angle between mandibular plane (ML) and occlusal functional plane (Oc) to evaluate the orientation of the superior maxilla; NL/Fh, angle between superior maxilla (NL) and Frankfurt plane (Fh) to evaluate the orientation of the superior maxilla.

Linear measurements are: ArGo, length of the ramus of the mandible; ML, length of the mandibular body; Wits index, distance between point A and point B projection on occlusal plane, to evaluate jaws disharmony. The values considered on cephalometry are shown in Fig. 1.

To quantify asymmetries between the mandibular condyles and the rami, the method introduced by Habets et al. [24] was used. This method compared the vertical heights of the mandibular right and left condyles and rami. Panoramic radiographs were traced and measured with a digital calliper by one operator blinded to the group division. The contours of the condyle and ascending ramus of both sides were accurately traced. On the tracing paper, a line (A, the ramus tangent) was drawn between the most lateral points of the condylar image (O1) and of the ascending ramus image (O2). A line perpendicular (B) to the ramus tangent was drawn from the most superior point of the condylar image. The vertical distance on the ramus tangent from the B line to the most lateral point of the condyle (O1), called condylar height (CH), and the distance on the ramus tangent between the two originally marked most lateral points of the image (O1 and O2), called ramus height (RH), were measured. To assess the symmetry between the condyles and the rami on the panoramic X-ray, the following formula |(RL)/(R7L)| 100% was used. The absolute value of the difference between the measured condylar or rami sizes of the right (R) and left (L) were divided by the sum of the same condylar or rami sizes and respectively expressed in percentages. This calculation allows individual differences in sizes and provides a value for (a) symmetry of each individual. The result of this ratio formula gives a range of asymmetry from 0% (complete symmetry) to 100%. A value of 6% difference between the condylar vertical sizes in a panoramic X-ray was considered an acceptable limit to diagnose condylar asymmetry [24].

The primary outcome of this study was the evaluation of the condylar asymmetry together with the divergency of the skull represented by the angles NSL/ML; NL/ML; NL/F and the linear measurement AR:GO.

### Method error

Eighteen lateral cephalograms were randomly selected choosing the 9 youngest patients and controls, traced and measured twice in a week interval by one operator (RC). The error was calculated with the interclass correlation coefficient (ICC) and with Springate’s method of moments variance estimator (MME).

The sample size calculation was done considering the prevalence of RA and the RA patients requiring orthodontic therapy. A sample size of 12 patients was required.

### Statistical analysis

Data are reported as the mean ± SD or the median (interquartile range). The statistical distribution of the quantitative measures was non-Gaussian (tested by the Shapiro–Wilk test), and we used the Mann–Whitney test to assess the significance of between-group differences. All the tests were two tailed, and the statistically significant level was set at 5%.

## Results

**Fig. 2:**
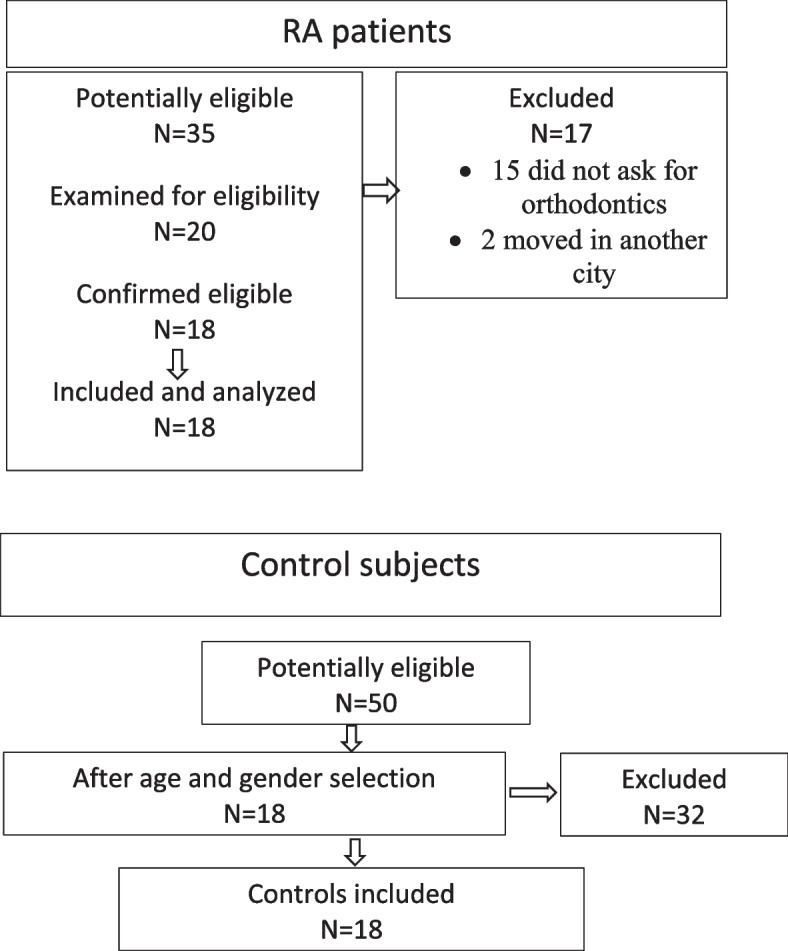
Flowchart of included patients

Out of 35 patients with a confirmed diagnosis of RA considered for this study, 18 patients (15 females and 3 males) were included. The control group was composed of 18 subjects (14 females and 4 males) (Fig. 2).

Demographic characteristics, and dental occlusion are presented in Table 1.

**Table 1:** Demographic and occlusal features of RA patients and control subjects

	Patients			Control		
	Males	Females	Total	Males	Females	Total
Number	3 (16.7%)	15 (83.3%)	18 (100%)	4 (22.2%)	14 (77.8%)	18 (100%)
Age at OPT (years)	57.7 ± 8.1	57.3 ± 12.2	57.4 ± 11.4	33.5 ± 6.2	34.7 ± 11.9	34.4 ± 10.8
**Type of Occlusion**						
Class I	1(33,3%)	7(46,6%)	8(44,4%)	2(50%)	8(57,1%)	10(55,6%)
Class II	0	5(33,4%)	5(27,8%)	2(50%)	5(35,7%)	7(38,8%)
Class III	1(33,3%)	1(6,6%)	2(11,2%)	0	1(7,2%)	1(5,6%)
Asymmetric	1(33,3%)	2(13,4%)	3(16,6%)	0	11(7,2%)	1(5,6%)
**Crossbite**						
Dx	0	3(20%)	3(16,7%)	0	0	0
Sx	0	1(6,6%)	1(5,6%)	0	0	0
Bilateral	0	0	0	0	0	0
No Crossbite	3(100%)	11(73,4%)	14(77,7%)	4(100%)	14(100%)	18(100%)

The angular and linear measurements of cephalometry did not show any significant difference except for the steepness of the occlusal plane (NL/OC), which was lower for patients (*P* < 0.02) and the vertical ramus of the mandible (Ar:Go) which was higher for patients.

Regarding condylar asymmetry, the results showed a significant difference in the range of asymmetry of the condyle, with the patient group being asymmetrical (*P* < 0.003). No differences were found in the range of asymmetry of the ramus between groups (*P* = 0.78) (Table 2).

**Table 2:** Comparison between groups of cephalometry and percent difference of right and left condyles and mandibular rami (*P* < 0.05)

	Patients (*n* = 18)	Controls (*n* = 18)	p
Female	15 (83.3%)	14 (77.8%)	
Age at OPT (years)	57.4 ± 11.4	34.4 ± 10.8	
Angular Measurements (°)			
SNA	80.7 ± 3.9	80.4 ± 3.7	0.87
SNB	78.0 ± 4.8	77.8 ± 4.0	0.90
ANB	2.6 ± 3.3	2.7 ± 3.4	0.92
SN^∧^GoMe(NSL/ML)	32.2 ± 6.7	31.2 ± 7.2	0.69
F^∧^GoMe(Fh/ML)	20.9 ± 6.6	20.3 ± 6.2	0.81
SpP^∧^GoGn(NL/ML)	23.1 ± 6.0	24.3 ± 6.2	0.57
NL/Oc	8.0 ± 3.0	10.7 ± 2.6	0.02
ML/Oc	16.4 ± 5.8	13.3 ± 5.3	0.16
NL/Fh	1.8 ± 4.2	3.8 ± 4.8	0.08
ArGo/ML	120.1 ± 6.4	116.7 ± 6.6	0.14
Linear Measurements (mm)			
ArGo	66.4 ± 8.7	49.6 ± 7.1	0.001
ML	94.3 ± 7.1	88.4 ± 11.0	0.03
Witts	0.5 ± 3.3	-0.8 ± 3.5	0.37
Asymmetry Index			
Condilar (CI)	10.6 ± 3.9	5.8 ± 4.8	0.003
Ramal (RI)	3.7 ± 2.5	6.7 ± 11.9	0.78

## Discussion

This study aimed to evaluate the cranial structure together with the condylar asymmetry of patients diagnosed with RA during adulthood, after 25 years of age, compared with normal adult subjects. To the best of our knowledge there are no previously published data in the literature on this topic in adult patients.

As expected, the results regarding angular and linear measurements in cephalometry showed a difference between RA patients and the control group only for the steepness of the occlusal plane (NL/OC) and the higher length of the vertical ramus of the mandible (Ar:Go) (*P* < 0.003); the condyles were different between patients and controls being asymmetrical in patients.

Regarding the bone features, the higher length of the vertical ramus of the mandible of RA patients indicates that during the growth period the development of the joint was not affected by the severe systemic inflammation. A previous study with the same research plan, was conducted on young patients diagnosed with juvenile idiopathic arthritis (JIA). Interestingly JIA patients showed a significant difference regarding the majority of the measurements of the skull leading to a typical hyperdivergent cranial structure (NSL/ML, NL/ML) with a serious backwards rotation of the mandible (Fh/ML), short vertical ramus (Ar:Go), steep mandibular plane (ML/Oc) [23]. None of those values was significantly different between RA patients and the control group. The features of the JIA patients are likely due to the insufficient growth of the condylar site and vertical ramus which are exposed to the severe inflammatory processes during development. It is intriguing to underline that RA patients did not show any significant difference in the cephalometric values compared to the control group; the length of the vertical ramus, which was shorter in JIA patients, was longer in RA patients meaning that the growth occurred according to individual genetics.

Regarding the condyles, RA patients showed a significant asymmetry between sides and a significant difference from the control group. This indicates that the systemic inflammation even in adults involves the temporomandibular joint which undergoes continuous functional work, load and forces maintaining compensatory capabilities lifelong. Interestingly, the condylar asymmetry of JIA children previously evaluated was much more severe than RA patients (percent difference of right and left condyles in JIA children 18.1 ± 9.5 [23] and in RA patients 10.6 ± 3,9. The percent difference of right and left vertical ramus of the mandible was not different in both groups.

The results confirmed the hypothesis of this work i.e. when RA is diagnosed in adulthood at the end of the cranial growth without the influence of pathological inflammation during development, the alterations of the skull previously shown in young JIA patients [19–23], are not present. Differently the asymmetry of the condyle has been also shown in RA patients because it is an adaptable structure aimed at maintaining a valid function [25]. This capability decreases in adulthood even if a higher turnover is maintained with respect to the other joints of the body.

**Fig. 3:**
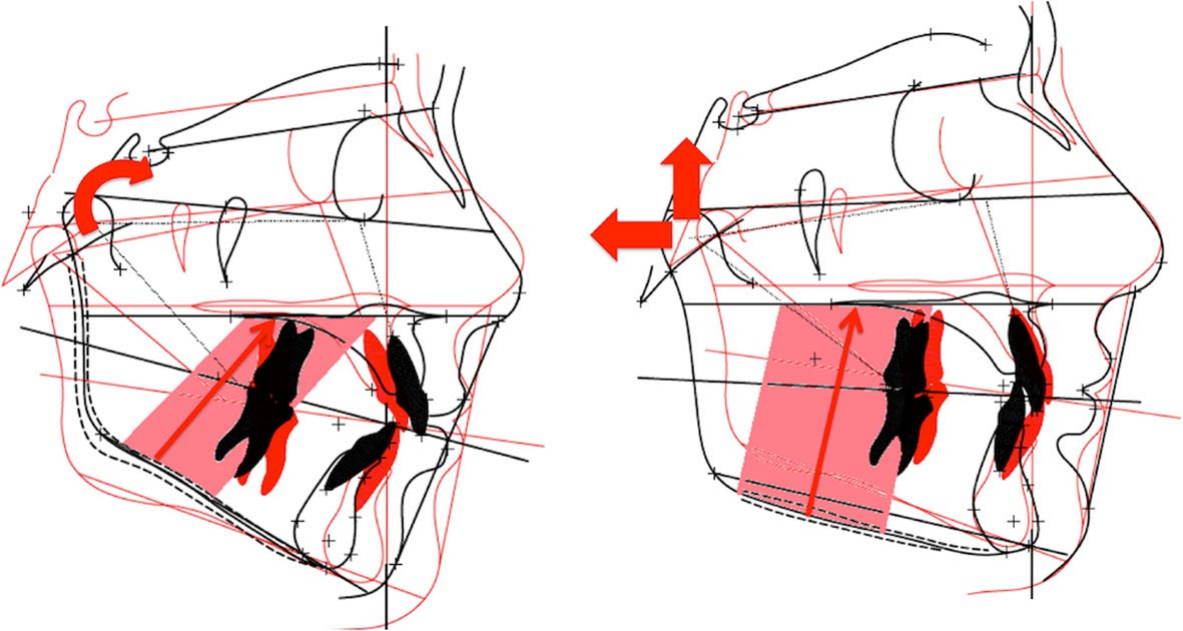
Comparison of the cranial structure of a JIA and a RA patient. Panel **A** image of the right profile of the skull of a JIA patient with unfavorable steepened vectors of the muscles and temporo-mandibular joint [23]. Panel **B** Image of the skull of an RA patient showing favorable straight muscular and temporomandibular joint vectors

The clinical outcome of these results underlines the importance of always diagnosing the TMJ pathology in conditions of arthritis: signs and symptoms that are usually not reported by the patients are to be carefully searched in the history and if necessary with instrumental exams. In addition, TMJ assessment needs to be updated over time, knowing that the TMJ supports daily unavoidable loads and forces. This is due to the functional and anatomical features of this joint strictly related to the vectorial features of the cranial structure (Fig. 3) [26–30]

The therapeutic choice, both during growth and adulthood, should be based on gnathological principles with respect to the physiology of functions.

The limitations of this study are related to the limited number of patients and controls. However, the homogeneous results led us to conclude that RA patients show an individual growth pattern not affected by inflammation, but clear joint asymmetry.

## Data Availability

The datasets used and/or analysed during the current study available from the corresponding author on reasonable request.
